# Associations between scores of psychosomatic health symptoms and health-related quality of life in children and adolescents

**DOI:** 10.1186/1477-7525-11-176

**Published:** 2013-10-23

**Authors:** Pia Svedberg, Mårten Eriksson, Eva Boman

**Affiliations:** 1 Division of Insurance Medicine, Department of Clinical Neuroscience, Karolinska Institutet, Stockholm, Sweden; 2 Faculty of Health and Occupational Studies, Department of Social Work and Psychology, University of Gävle, Gävle, Sweden

**Keywords:** Health, Quality of life, Gender, School, Children, Adolescents, Psychosomatic symptoms, Sleep problems, KIDSCREEN

## Abstract

**Background:**

The aims of the present study are to investigate whether there are differences in health-related quality of life (HRQoL) between girls and boys in two different age groups, to study how much of children’s variance in HRQoL can be explained by common psychosomatic health symptoms, and to examine whether the same set of psychosomatic symptoms can explain differences in HRQoL, both between girls and boys and between older and younger school children.

**Methods:**

A cross-sectional study was conducted of 253 children, 99 of ages 11–12 years (n=51 girls, n=48 boys) and 154 of ages 15–16 years (n=82 girls, n=72 boys), in Swedish schools. The KIDSCREEN-52 instrument, which covers 10 dimensions of HRQoL and additional questions about psychosomatic health symptoms, were used. Analyses of variance were conducted to investigate differences between the genders and age groups, and in interaction effects on the KIDSCREEN-52 dimensions. Regression analyses were used to investigate the impacts of psychosomatic symptoms on gender and age group differences in HRQoL.

**Results:**

Boys rated themselves higher than girls on the KIDSCREEN dimensions: physical and psychological well-being, moods and emotions, self-perception, and autonomy. Main effects of age group were found for physical well-being, psychological well-being, moods and emotions, self-perception, autonomy, and school environment, where younger children rated their HRQoL more highly than those aged 15–16 years. Girls rated their moods and emotions dramatically lower than boys in the older age group, but the ratings of emotional status were more similar between genders at younger ages. Psychosomatic symptoms explained between 27% and 50% of the variance in the children’s HRQoL. Sleeping difficulties were a common problem for both girls and boys. Depression and concentration difficulties were particularly associated with HRQoL among girls whereas stomach aches were associated with HRQoL among boys.

**Conclusions:**

Girls and adolescents experience poorer HRQoL than boys and younger children, but having psychosomatic symptoms seem to explain a substantial part of the variation. Strategies to promote health among school children, in particular to alleviate sleep problems among all children, depression and concentration difficulties among girls, and stomach aches among boys, are of great importance.

## Background

Health-related quality of life (HRQoL) in children and adolescents has received increased attention in recent years, and is now an emerging area of research [1,2]. Some studies have indicated that both physical and mental health have deteriorated among school children over the past decades [3,4]. Measures of HRQoL have therefore become increasingly important, in studies not only of chronically ill children [1,2], but also among children in general, about whom knowledge is still limited. Studies of HRQoL can have considerable significance for understanding children’s psychosocial functioning, and their perceptions of illness and disease [5,6]. Some population-based studies have reported gender differences in HRQoL among children, adolescents and adults [7-9]. However, these results need to be replicated in different cultural settings, and there is also a need for more detailed studies of the specific dimensions related to reported gender discrepancies.

Information from generic HRQoL measures for children and adolescents are useful in identifying subgroups of children and adolescents at risk of health problems [1,7,10]. HRQoL assessments incorporate not only the impacts of disease and treatment on physical functions, but also their effects on lifestyle and emotional well-being. HRQoL deals with the impact of functional impairment on all aspects of life, including the ability of children to go to school or play, and also the emotional effects of any such functional impairment. A previous study [11] showed that perceived health, school connectedness, health promotion, bullying, visits to school nurse, and age significantly predicted 44% of the variation in HRQoL among girls. Higher family wealth was found to be positively associated with greater physical well-being [10,12]. These associations, detectable as early as at age 8 [12], might have implications for children’s physical and mental health in adolescence or early adulthood. Gender differences in HRQoL and health perceptions, including health symptoms, have been observed in previous studies of general child populations [3,4,7,9-11,13-15]. Most studies report that boys and girls have similar HRQoL at young ages. However, gender differences are observable in adolescence, where there is a more pronounced decline in HRQoL among girls [7,10,15], on both mental and physical dimensions [10]. Similar developmental trajectories have been observed for related measures of health perceptions, such as self-rated health and general well-being [3,4]. However, most of the previous studies of HRQoL that have included health symptoms have been directed at particular patient groups; for example a recent study reports that children with average or severe mental-health problems have lower psychosocial and physical HRQoL scores than children in general [16]. Less is known about various psychosomatic health symptoms in general populations, and their associations with HRQoL. It is therefore of interest further to investigate whether also common psychosomatic symptoms, such as sleep problems, concentration difficulties and milder depressive symptoms, are associated with various HRQoL dimensions, at different ages and between the genders.

The aims of the present study are to investigate whether there are differences in HRQoL between girls and boys, and between children in two age groups (11–12 and 15–16 years), and also whether there are any interaction effects of gender and age group on different dimensions of HRQoL. Further aims were to study how much of children’s HRQoL can be explained by psychosomatic health symptoms, and to investigate whether the same set of symptoms can explain the differences found in HRQoL between girls and boys, and between younger and older school children. In line with previous findings [7,9,10,15], we expected to find gender and age-group differences in HRQoL.

## Methods

### Sample and data collection

A cross-sectional study was conducted of 283 children at school in Sweden, with data on HRQoL and health symptoms collected in 2009. The questionnaire, which took approximately 20 minutes to fill out, was administered in school classrooms and filled out individually by the children. School classes were selected from Stockholm, the capital city of Sweden, and a mid-sized Swedish town. Schools from both lower and higher socioeconomic areas of living in both places were included in order to assure a sample with variation in socioeconomic status (SES) and urbanity. In total 13 classes from seven schools were involved. Informed consent was collected from the guardian, but the child had also the possibility to refrain participation. External dropout was 10%, due to sickness absence the day the questionnaire was administered. The sample comprised 253 participants, that is 99 participants of ages 11–12 years (51 girls and 48 boys) from grades 5, and 154 participants of ages 15–16 years (82 girls and 72 boys) from grade 9. This study was approved by the regional ethical committee board, Uppsala, Sweden (Dnr 2009/021, date: 2009-05-13).

### Measures

#### Health-related quality of life

HRQoL was measured using the KIDSCREEN-52 questionnaire, which is a generic self-report instrument designed for use among children and adolescents that takes 10–20 minutes to complete [9,17]. The KIDSCREEN-52 measures HRQoL on 10 dimensions: physical well-being (5 items); psychological well-being (6 items); moods and emotions (7 items); self-perception (5 items); autonomy (5 items); parent relations and home life (6 items); social support and peers (6 items); school environment (6 items); social acceptance and bullying (3 items); and, financial resources (3 items). Responses were given on a 5-point Likert-type scale, and ranged from 'never’ to 'always’ to assess frequency, and from 'not at all’ to 'extremely’ to assess intensity during the past week. Following the KIDSCREEN manual, Rasch scores were computed for each dimension, weighted in accordance with the European norm population, and reported as T-values [9,17]. Higher scores indicate better HRQoL. Further, a composite general HRQoL index was computed by selecting 10 dimensions that jointly give an overall HRQoL score. It has been shown previously that the instrument has high reliability, validity and internal consistency [17-19]. The Swedish version of the questionnaire has demonstrated acceptable validity and reliability. A more detailed description of the KIDSCREEN instrument and its items can be found elsewhere [9,17,19].

#### Self-reported psychosomatic health symptoms

Frequencies of health symptoms during the past school year were measured using eight items from the checklist in the WHO study, Health Behaviour in School-Aged Children (HBSC) from the 1980s [20,21], which has been used and validated in several studies [22,23]. The eight items have later also been used as a scale (sometimes referred to as the Psychosomatic Problems scale (PSP) and its’ psychometric properties has been carefully described elsewhere [24-26]. PSP consists of the following items: Difficulty Concentrating, Sleep problems, Headache, Stomach Ache, Tensions, Lack of appetite , Depressive symptoms (felt low), and Dizziness. Response alternatives were on a 5-point Likert-type scale, ranging from 'never’ to 'always' during the current school year. The psychosomatic symptoms (PSP-scale, alpha coefficient = .87) was used as a covariate.

### Statistical analyses

Analyses of variance (ANOVA) were conducted in order to investigate differences between genders, age-group and interaction effects in relation to the KIDSCREEN-52 dimensions and the composite HRQoL item. A multivariate analysis (MANOVA) was performed with gender and age-group as independent variables and the 10 KIDSCREEN dimensions as dependent variables. The PSP-scale was used as a covariate in the same type of ANOVA as described above, with gender and age group as independent variables and composite general HRQoL as the dependent variable. In order to establish whether gender and age-group differences can be explained by health symptoms, a series of stepwise regression analyses were conducted.

## Results

Of the 253 responders, 8% had missing information on one or more single KIDSCREEN items, and three individuals had missing information on one or more single psychosomatic health symptom items. Internal missing data on any single item was imputed on the basis of the modal value of that specific item.

In the MANOVA an overall effects were found for gender (Wilks *λ=*0.78, *F*(10,240)=6.44, *p*<0.01), for age group (Wilks *λ=*0.85, *F*(10,240) =4.07, *p*<0.01), and for the interaction between gender and age group (Wilks *λ=*0.91, *F*(10,240)=2.33, *p*=0.01).

An analysis of variance (ANOVA) of the composite general HRQoL showed an overall mean of 51.06 (95% CI 50.09-52.03). Boys (*M*=52.03, *SD*=0.71) scored higher than girls (*M*=50.09, *SD*=0.68, *F*(1,249)=3.92, *p*=0.049), and children aged 11–12 (*M*=52.35, *SD*=0.77) scored higher than 15–16 year-old adolescents (*M*=49.77, *SD*=0.62, *F*(1,249)=6.87, *p*<0.01).

### Gender and age group differences on each HRQoL dimension

The follow-up ANOVAs on the KIDSCREEN HRQoL dimensions showed five main effects of gender: on Physical well-being, Psychological well-being, Moods and emotions, Self-perception, and Autonomy. Boys rated higher than girls on all these dimensions (Physical well-being, boys *M*=49.06, *SD*=11.82, girls *M*=45.01, *SD*=8.25; Psychological well-being, boys *M*=53.38, *SD*=11.51, girls *M*=49.85, *SD*=11.65; Moods and emotions, boys *M*=52.49, *SD*=12.24, girls *M*=44.81, *SD*=13.29; Self-perception, boys *M*=52.38, *SD*=11.82, girls *M* = 46.87, *SD*=11.71; and Autonomy boys *M* = 49.76, *SD* = 11.49, girls *M* = 45.53, *SD* =12.24). See Table 1.

**Table 1:** **Results of the MANOVA on the KIDSCREEN dimensions among Swedish school children, main and interaction effects of by gender and age group (11–12 and 15–16 years), and effect sizes (η**^**2**^**)**

**KIDSCREEN dimension**	**Variable**	**df**	***F***	***p***	**η**^**2**^
Physical well-being	Gender	1	6.903	**0.009**	0.027
	Age group	1	4.216	**0.041**	0.017
	Gender*Age group	1	5.245	**0.023**	0.021
	MS error	249	98.926	-	-
					
Psychological well-being	Gender	1	5.285	**0.022**	0.021
	Age group	1	5.454	**0.020**	0.021
	Gender*Age group	1	0.034	0.853	0.000
	MS error	249	132.275	-	-
					
Moods and emotions	Gender	1	16.956	**0.000**	0.064
	Age group	1	0.111	0.740	0.000
	Gender*Age group	1	6.993	**0.009**	0.027
	MS error	249	160.651	-	-
					
Self-perception	Gender	1	12.935	**0.000**	0.049
	Age group	1	9.531	**0.002**	0.037
	Gender*Age group	1	0.040	0.842	0.000
	MS error	249	134.202	-	-
					
Autonomy	Gender	1	5.957	**0.015**	0.023
	Age group	1	22.135	**0.000**	0.082
	Gender*Age group	1	2.802	0.095	0.011
	MS error	249	129.195	-	-
					
Parent relations and home life	Gender	1	0.035	0.853	0.000
	Age group	1	1.562	0.213	0.006
	Gender*Age group	1	1.545	0.215	0.006
	MS error	249	147.530	-	-
					
Peers and social support	Gender	1	0.085	0.771	0.000
	Age group	1	0.354	0.552	0.001
	Gender*Age group	1	0.442	0.507	0.002
	MS error	249	146.247	-	-
					
School environment	Gender	1	1.488	0.224	0.006
	Age group	1	15.685	**0.000**	0.059
	Gender*Age group	1	0.731	0.393	0.003
	MS error	249	125.425	-	-
					
Social acceptance and bullying	Gender	1	2.176	0.141	0.009
	Age group	1	0.477	0.490	0.002
	Gender*Age group	1	0.832	0.363	0.003
	MS error	249	146.816	-	-
					
Financial resources	Gender	1	1.947	0.164	0.008
	Age group	1	0.005	0.944	0.000
	Gender*Age group	1	0.021	0.885	0.000
	MS error	249	99.922		

*Note: p*-values in bold indicate statistical significance (*p* < 0.05).

Further, there were five main effects of age group: on Physical well-being, Psychological well-being, Self-perception, Autonomy, and School environment. Eleven to twelve year-old children scored higher on all five dimensions (Physical well-being 11–12 years *M*=48.65, *SD*=8.35, 15–16 years *M*=45.82, *SD*=11.24; Psychological well-being 11–12 years *M*=53.67, *SD*=10.20, 15–16 years *M*=50.14, *SD*=2.39; Self-perception 11–12 years *M*=52.36, *SD*=11.76, 15–16 years, *M*=47.64, *SD*=11.92; Autonomy 11–12 years *M*=51.84, *SD*=8.32, 15–16 years *M*=44.77, *SD*=13.24; and School environment 11–12 years *M*=53.05, *SD*=10.20, 15–16 years; *M*=47.30, *SD*=11.79). See Table 1.

**Figure 1:**
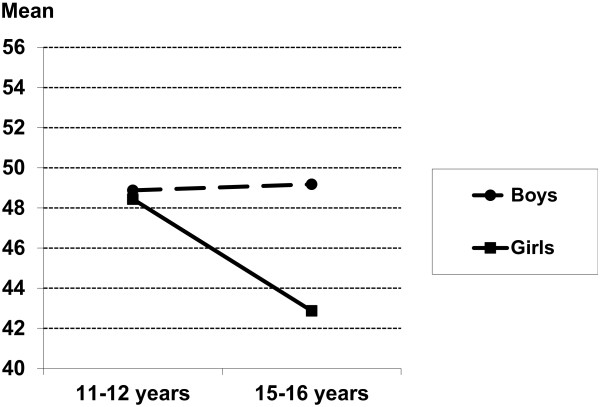
Interaction between gender and age group on the HRQoL Physical Well-Being dimension (Girls, age-group 11–12 years: mean=48.44, 95% Confidence Intervals (CI) 45.70-51.19; age-group 15–16 years: mean=42.87, 95% CI 40.71-45.03; Boys, age-group 11–12 years: mean=48.88, 95% CI 46.05-51.70, age-group 15–16 years: mean=49.18, 95% CI 46.87-51.48).

**Figure 2:**
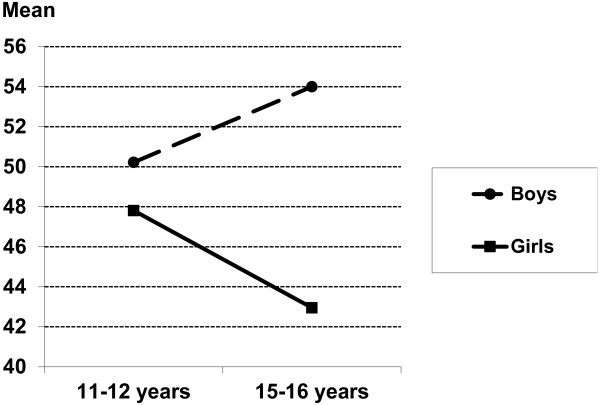
Interaction between gender and age group on the HRQoL Moods and Emotions dimension (Girls, age-group 11–12 years: mean=52.16, 95% Confidence Intervals (CI) 48.98-55.33; age-group 15–16 years: mean=48.42, 95% CI 45.91-50.92; Boys, age-group 11–12 years: mean=55.29, 95% CI 52.02-58.56, age-group 15–16 years: mean=52.10, 95% CI 49.43-54.77).

Two interaction effects of gender and age group on the HRQoL KIDSCREEN dimensions were found. Boys and girls rated their Physical well-being similarly at young ages (11–12 years). Among adolescents 15–16 years of age, boys rated their Physical well-being at the same level as did 11–12 year-old boys, whereas girls rated their Physical well-being substantially lower than did 11–12 year-old girls and boys (Figure 1). The second interaction was found on the Moods and emotions dimension. Boys aged 15–16 years rated their Moods and emotions higher than did younger boys, while the opposite pattern was found for girls (Figure 2). In general, effect sizes (eta^2^) were small to moderate. See Table 1.

### Psychosomatic symptoms and HRQoL – gender and age-group differences

When the psychosomatic symptoms (PSP) scale was used as a covariate in the ANOVA, with gender and age group as independent variables and composite general HRQoL as the dependent variable, neither gender nor age group, nor the interaction between them, contributed significantly to explaining the variance (all three F-values <1). It was therefore concluded that differences between boys and girls, and between age groups, are associated with the symptoms on this index.

From the series of step-wise regression analyses to further investigate the associations between psychosomatic symptoms and composite HRQoL, the psychosomatic symptoms were found to be moderately correlated with each other (with coefficients ranging from .34-.61), which is well below the .80 that is regarded as critical for multi-collinearity. First, a stepwise regression analysis (PIN=.05, POUT=.10) with Difficulty concentrating, Sleep problems, Headache, Stomach ache, Tensions, Lack of appetite, Depressive symptoms, and Dizziness as independent variables showed that a three-step model, with Sleep problems, Depressive symptoms and Difficulty concentrating in that order, explained 37% of the variance in HRQoL. See Table 2. Then, the same type of regression analysis was performed separately for girls and boys in a second and a third analysis. This resulted in a four-step model for girls, comprising Tensions, Depressive symptoms, Sleep problems, and Difficulty concentrating in that order, that explained 50% of the variance in HRQoL. See Table 2. For boys, a two-step model, comprising Sleep problems and Stomach ache, explained 27% of the variance in HRQoL. See Table 2.

**Table 2:** Results of stepwise regression analyses of psychosomatic health symptoms as predictors of HRQoL among Swedish school children

**Analyses**	***B***	***SE B***	***β***	***R***^***2***^
1st analysis (N=252)				.368
Step 1: Sleep problems	2.000	.403	.299	
Step 2: Depression (felt low)	2.108	.392	.301	
Step 3: Difficulty concentrating	1.284	.459	.170	
2nd analysis: Girls (N=132)				.503
Step 1: Tensions	1.509	.523	.227	
Step 2: Depression (felt low)	2.044	.489	.300	
Step 3: Sleep problems	1.296	.467	.206	
Step 4: Difficulty concentrating	1.469	.579	.198	
3rd analysis: Boys (N=119)				.272
Step 1: Sleep problems	3.051	.583	.434	
Step 2: Stomach ache	1.487	.666	.185	
4th analysis: 11–12 years (N=98)				.492
Step 1: Sleep problems	2.882	.572	.413	
Step 2: Lack of appetite	1.883	.570	.268	
Step 3: Depression (felt low)	1.651	.578	.232	
5th analysis: 15–16 years (N=153)				.319
Step 1: Depression (felt low)	2.252	.526	.330	
Step 2: Sleep problems	1.482	.519	.222	
Step 3: Difficulty concentrating	1.198	.604	.159	

*Note.* Regression coefficients are reported at the final step.

A three-step model, comprising Sleep problems, Lack of appetite and Depressive symptoms, explained 50% of the variance in HRQoL for 11–12 year-old children. For adolescents (15–16 years), a three-step model, comprising Depressive symptoms, Sleep problems and Difficulty concentrating, explained 32% of the variance in HRQoL. See Table 2.

Thus, psychosomatic health symptoms explained between 27% and 50% of the variance in the children’s HRQoL. Sleep problems had a major negative impact on all children’s HRQoL, but Depressive symptoms and Difficulty concentrating also had substantial impacts. Tensions, Depressive symptoms and Difficulty concentrating were particularly strongly associated with HRQoL among girls, whereas Stomach ache was associated with HRQoL among boys. Lack of appetite was associated with HRQoL at ages 11–12 but not at ages 15–16, whereas Difficulty concentrating was associated with HRQoL at ages 15–16 but not at ages 11–12.

## Discussion

This study investigated differences in HRQoL between girls and boys in two age groups. Boys scored higher than girls on the following dimensions: physical well-being, psychological well-being, moods and emotions, self-perception, and autonomy. Children aged 11–12 years reported higher levels of physical well-being, psychological well-being, self-perception, autonomy, and school environment than older children (15–16 years-old). These gender and age-group differences among Swedish adolescents are in line with previous findings from European studies [7,9,10,15] which supports their generalibility for adolescence in western cultures. An interaction effect was found for physical well-being. Boys and girls gave similar ratings at ages 11–12 years; but, at ages 15–16, boys were at the same level as at ages 11–12, whereas girls’ ratings at ages 15–16 were substantially lower than those of girls aged 11–12 years and those of boys in both age groups. Further, adolescent boys (15–16 years) rated their moods and emotions more highly than did younger children (11–12 years), whereas the opposite was found for girls. Psychosomatic health symptoms were found to explain a large part of the variation in HRQoL.

Overall, the psychosomatic symptoms explained between 27% and 50% of the variance in the children’s HRQoL, and the higher explanatory percentages were for girls and younger children. Depressive symptoms and concentration difficulties affected the girl’s HRQoL, while sleep problems had a major impact on all the children’s HRQoL. Sleep problems have previously been associated with both somatic and psychiatric problems [27,28], and sleep problems in childhood also predict behavioural and emotional problems in adolescence. Earlier research has shown that the overlap between sleep and behavioural problems may be of great significance in developmental change [29]. Moreover, the correlations between sleep problems, depression and anxiety have been shown to increase significantly with age [29]. Persistent sleep problems in childhood may also be an early risk indicator of anxiety in adulthood [30]. An improvement in sleeping patterns or sleep quality may protect against poor HRQoL among children and adolescents.

In the present study, differences were found with regard to the psychosomatic symptoms that were the most important for HRQoL in girls and boys at different ages. Tensions, depressive symptoms and difficulty concentrating were particularly associated with HRQoL among girls, whereas stomach ache was associated with HRQoL among boys. Lack of appetite was associated with HRQoL at grade five (11–12 years), but not at grade nine (15–16 years), whereas difficulty concentrating was associated with HRQoL at grade nine but not at grade five. As expected, the results showed that girls and boys at ages 11–12 rated their HRQoL more highly, and more equally, than did adolescent boys and girls [7,10,15].

Psychological theories of children and adolescents relate individual development to social and cultural factors. Adolescents face a series of age-related and gender-specific challenges over the years as part of the developmental process. Pubertal development involves physical maturation, body image, peer relationships, sexuality, and autonomy. This development is known to start at an earlier age in girls and gender differences in pubertal development might therefore explain the differences between girls and boys reported in the present study with regard to both level of HRQoL and psychosomatic symptoms. A future study involving a third assessment some years later would provide information on whether the assessed differences are merely a question of puberty onset. However, puberty in girls and boys also involves different processes including discharge of different hormones. Particularly the onset of menstruation is a common cause of many complaints and it has been reported that among female adolescents between 12 and 16 years of age these are among the most common health problem [7]. It is therefore likely that pubertal processes are responsible for the different levels of HRQoL and psychosomatic symptoms in this study. To gather information on pubertal markers such as menstruation in girls and lowered voice in boys together with assessments of HRQoL in future studies would therefore be desirable since it is still unclear whether pubertal developmental processes explain HRQoL differences between genders and age groups [31]. Another possibility is that the decrease in HRQoL, particularly among 15–16 years old girls, is associated to cultural norms and values in western culture. Future studies including non-western societies would shed light on this issue.

Studies of HRQoL may be of considerable significance in understanding children and adolescent’s psychosocial functioning and development, and also their perceptions of illness and disease and the effects of these on daily life [5,6]. The present study showed that HRQoL is associated to several psychosomatic symptoms. Sleeplessness was one common symptom and it is perhaps a good start for interventions to improve the health of young individuals. Regular routines, minimal exposure to electronic media and avoidance of caffeine at bedtime, as well as exercise are well-known practices consistent with good sleep hygiene [32].

### Strengths and limitations

Strengths of the present study include the use of a validated questionnaire on HRQoL, use of validated psychosomatic health items (PSP-scale), and the administration to general child populations in various socioeconomic areas (Swedish municipalities). There were low rates of both external and internal dropout. Also, the children themselves were asked about their HRQoL, rather than relying on proxy reports by parents. Limitations include the rather small sample size and a lack of more objective health measures, such as those obtainable from physical examinations or medical records, and household socioeconomic status. Further, given the cross-sectional design we were unable to evaluate the direction of association between psychosomatic symptoms and HRQoL.

## Conclusion

In sum, girls and adolescents report poorer HRQoL than boys and younger children, respectively, and psychosomatic symptoms seem to explain a substantial part of the variation in HRQoL. Finding strategies to promote health among school children, in particular to alleviate sleep problems among all children, depression and concentration difficulties among girls, and stomach aches among boys, seem to be of great importance.

## Competing interests

The authors declare that they have no competing interests.

## Authors’ contributions

PS and EB designed the study. PS, EB and ME conducted the statistical analyses. PS and EB drafted the first version of the manuscript and PS led its critical review. All the authors contributed substantially to the interpretation of results, the drafting of the text, and approved the final manuscript.
